# Three-dimensional motion control of an untethered magnetic object using three rotating permanent magnets

**DOI:** 10.1038/s41598-023-45419-2

**Published:** 2023-10-23

**Authors:** Hiroshi Sakuma

**Affiliations:** School of Engineering, Utsunomiya University, 7-1-2 Yoto, Utsunomiya, 321-8585 Japan

**Keywords:** Mechanical engineering, Electrical and electronic engineering, Biomedical engineering

## Abstract

Magnetic motion control has been actively studied mainly for the purpose of biomedical applications. However, in many cases, many actuator magnets surround a small magnet to be moved, and they consume large electric power. In some cases, complex calculations are required to estimate the control input of the actuator magnets. This study proposes a simple method to move a small magnet to the desired positions. For this, three cylindrical permanent magnets magnetized in the radial direction were positioned as the sides of a triangle; these actuator magnets were rotated using motors. By monitoring the position of the small magnet and through simple feedback control based on the angles of the three actuator magnets, the untethered small magnet could be moved along arbitrary three-dimensional (3D) paths. The control principle was established by calculating the magnetic force and torque acting on the small magnet for some sets of actuator-magnet angles.

## Introduction

Most are familiar with experiments that demonstrate how paper clips or iron particles are attracted by a magnet. The magnetic field around the magnet rotates the magnetic moment of the clips and iron particles, which are then attracted to the magnet within the field gradient. Conversely, it is typically difficult to push these items away from the magnet^1^ because the magnetic moment rotation must be prevented. The easiest way of moving them in a given direction is to set the magnet in the direction of movement. Thus, in many magnetic motion control systems, magnets surround magnetic objects. However, in biomedical applications such as capsule endoscopy^2^ and targeted drug delivery^1^, a magnetic field must be exerted over a large area, such as the human body. The magnetic field weakens steeply with increasing distance from the magnet. Therefore, it is favorable for the magnets to be positioned on one side^3^ with the opposite side being opened, which we refer to as an “open workspace.”

There is a lot of literature on magnetic motion control, which has been summarized in some reviews^1,2,4–7^. They are classified into two groups based on the control principle: gradient pulling and helical or sperm-like propulsion^6^. The former uses a magnetic force exerted on a magnetic object in a gradient field^3,8–14^. It is suitable for in-vitro applications because the direction and magnitude of force can be precisely controlled. The latter uses rotating or oscillating motion of helical or sperm-like magnets^11,15–18^. It is suitable for in-vivo applications because of the great propulsion force in a lumen such as the gastrointestinal system. This study focus on the gradient pulling considering the possibility of levitation in air.

Actuator magnets (magnetic-field sources) that are employed for motion control are classified into electromagnets and permanent magnets. Permanent magnets are energy efficient because they do not consume energy to generate a magnetic field. For example, a power supply of 6 kW is required to drive eight electromagnets^9^, whereas the power consumption of motors required to rotate eight permanent magnets^11^ is as small as 100 W. In addition, permanent magnets generate stronger magnetic fields and field gradients than those generated by electromagnets of the same size^4^. However, the number of demonstrations with permanent magnets is fewer than those using electromagnets because of their complexity: the magnetic field of a permanent magnet cannot be turned off similar to an electromagnet, and field manipulation is only possible through physical movement of the magnet^7^.

Mahoney et al.^10^ demonstrated 5 degrees-of-freedom (5-DOF) (position and orientation) control using only one permanent magnet. However, the magnet was mounted on a robotic arm capable of 6-DOF motion. Ryan et al.^11^ demonstrated 5-DOF control using eight rotating permanent magnets positioned on a sphere; however, they did not aim for open workspace. In both the studies, magnetic force and torque were calculated by approximating the permanent magnets as point dipoles. To propel the magnetic object in a desired direction, an inverse problem must be solved. In many applications, 3-DOF (only position) is sufficient. From a simple algebraic perspective, three magnets are sufficient for 3-DOF control^19^.

Supplementary Table S1 (see online) summarizes the reported demonstrations of motion control using permanent magnets and compares them with the present study. In this study, 3-DOF (position) control was demonstrated using a simple system equipped with three permanent magnets. The actuator magnets are rotated using stepper motors. An untethered magnetic object was moved along arbitrary 3-dimensional (3D) paths in an open workspace with the field gradient of the actuator magnets. No support for magnetic objects, such as a plate or Petri dish, which is used in 2-DOF position control, is required. In contrast to the aforementioned methods, the calculation or optimization of an inverse problem is not required. However, it is based on a simple feedback control. To the best of our knowledge, this study proposes the simplest method for achieving 3D magnetic motion control.

## Magnetic motion control system

**Figure 1:**
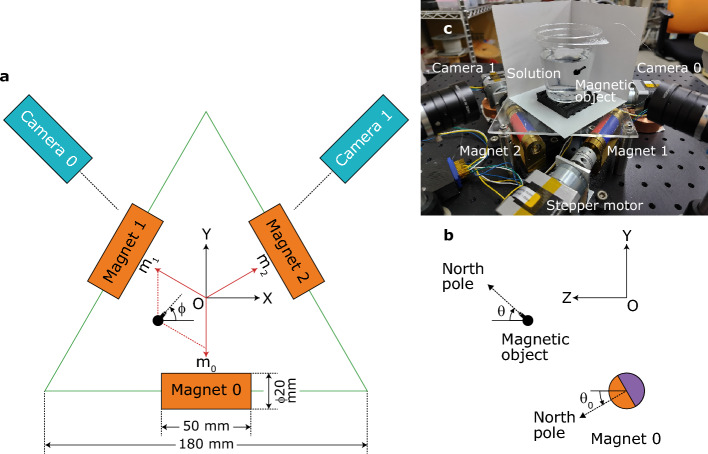
Schematic of the magnetic motion control system with magnet numbers, magnet angles, and coordinates of the magnetic object. (**a**) Top view and (**b**) side view [magnets 1 and 2, and cameras are not shown in (**b**) for clarity]. The angles of magnets 0–2 (*θ*_0_–*θ*_2_) are positive for outward inclination. *m*_0_–*m*_2_ are magnet coordinates obtained from the *XY* position of the magnetic object. (**c**) Photograph of the system viewed from + *Y* direction.

Figure 1 shows a schematic of the magnetic motion control system and the coordinates. The magnetic field is tuned using three cylindrical neodymium magnets magnetized in the radial direction. They can be rotated independently; however, their positions are fixed at the center of each side of an equilateral triangle. Hereafter, position (*X*, *Y*, *Z*) of the magnetic object will be expressed in millimeters. We use magnet angles of (*θ*_0_, *θ*_1_, *θ*_2_) = (0°, 0°, 0°) as a reference, which generates a magnetic field in the + *Z* direction at a point (*X*, *Y*, *Z*) = (0, 0, 55). The north pole of the magnetic object is aligned in the + *Z* direction.

## Finite element method simulations

We discuss the principle of motion control using finite element method (FEM) simulations. The magnetic object is a single magnet, which is small enough to be considered to have point magnetic moment. Force ***F*** acting on magnetic moment ***m*** in magnetic flux density ***B*** is as follows^20,21^:

1 $${\varvec{F}}=\nabla \left({\varvec{m}}\cdot {\varvec{B}}\right)$$

The magnetic object is moved according to this formula, which is called magnetic gradient pulling. In addition, torque ***T*** is considered as follows:

2 $${\varvec{T}}={\varvec{m}}\times {\varvec{B}}$$

**Figure 2:**
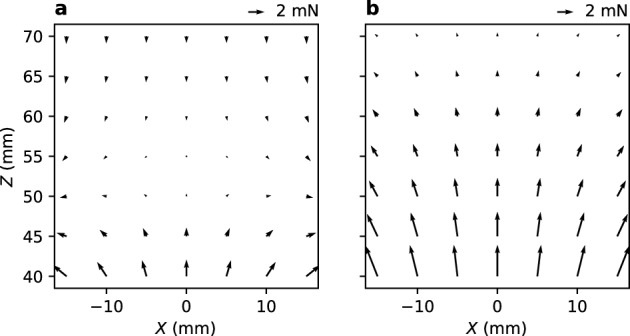
Magnetic force acting on the magnetic object placed in the *XZ* plane (*Y* = 0). Magnet angles (*θ*_0_, *θ*_1_, *θ*_2_) are (**a**) (–20°, –20°, –20°) and (**b**) (20°, 20°, 20°). Magnetic object is not inclined (*θ* = 0°).

First, the principle of control in the *Z* direction is discussed. Figure 2 shows the force acting on the magnetic object placed in the *XZ* plane for (*θ*_0_, *θ*_1_, *θ*_2_) = (–20°, –20°, –20°) and (20°, 20°, 20°). The maps of magnetic flux density are separately shown in Supplementary Fig. S1. The difference between gravity and buoyancy acting on the magnetic object is 0.9 mN (see “Methods”). Therefore, a magnetic force of 0.9 mN in the + *Z* direction will balance it; for this, magnet positions are found to be (*X*, *Y*, *Z*) = (0, 0, 47) and (0, 0, 64) for (*θ*_0_, *θ*_1_, *θ*_2_) = (–20°, –20°, –20°) and (20°, 20°, 20°), respectively. Thus, the *Z* position is controlled by rotating the three magnets in unison.

**Figure 3:**
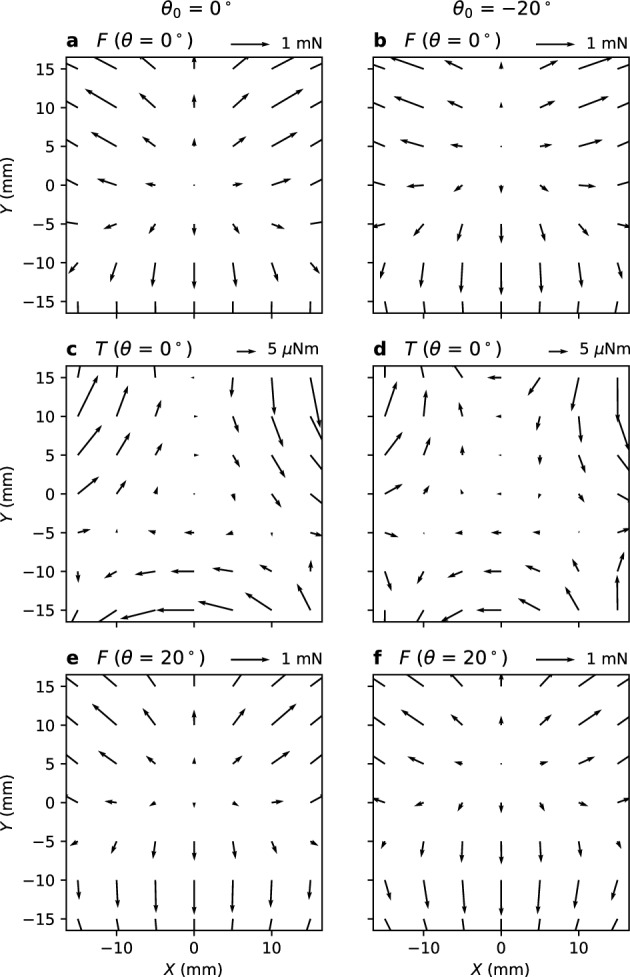
Magnetic force and torque acting on the magnetic object placed in the *XY* plane (*Z* = 55 mm). Magnet angles (*θ*_0_, *θ*_1_, *θ*_2_) are (0°, 0°, 0°) for left panels (**a**, **c**, **e**) and (–20°, 0°, 0°) for right panels (**b**, **d**, **f**). (**a**, **b**) Force and (**c**, **d**) torque without magnetic object inclination (*θ* = 0°). (**e**, **f**) Force for the same conditions as (a, b) with magnetic object inclination of *θ* = 20° and *ϕ* = 90°.

Control in the *XY* direction is a little complicated. Figure 3a shows the magnetic force acting on the magnetic object placed in the *XY* plane for (*θ*_0_, *θ*_1_, *θ*_2_) = (0°, 0°, 0°). The maps of magnetic flux density are separately shown in Supplementary Fig. S2. The *X* and *Y* components of the force acting on the magnetic object placed at *X* = *Y* = 0 are zero. As shown in Fig. 3b, by rotating magnet 0 such that *θ*_0_ = –20°, a force develops in the –*Y* direction. However, the stable point, at which the force in the *X* and *Y* directions is zero, moves in the + *Y* direction. Therefore, to position the magnetic object at the desired point, magnet 0 should be rotated in the opposite direction. Figure 3c and d show the torque under the same conditions as in (a) and (b), respectively. As the magnetic object moves in the –*Y* direction, a torque to incline the magnetic object to *ϕ* = 90° develops. As shown in Fig. 3e and f, this inclination enhances the force in the –*Y* direction. Thus, the *X* and *Y* positions are controlled by independently rotating the three magnets.

Magnet coordinates are introduced to move the magnetic object to an arbitrary *XY* position. The position vector is decomposed into *m*_0_–*m*_2_ components. For example, the position of the magnetic object shown in Fig. 1 is represented by (*m*_0_, *m*_1_, 0). To move the magnetic object from the center (*X* = *Y* = 0) to (*m*_0_, *m*_1_, 0), magnets 0 and 1 must be rotated in the negative direction. Although *m*_0_–*m*_2_ are always positive, the magnets can be rotated in any direction because feedback control is based on the difference between the setpoint (SP) and process value (PV). In this manner, propulsion in any direction is implemented without calculating magnetic force or torque. The detail of the feedback control is described in the “Methods” section.

## Experimental results

**Figure 4:**
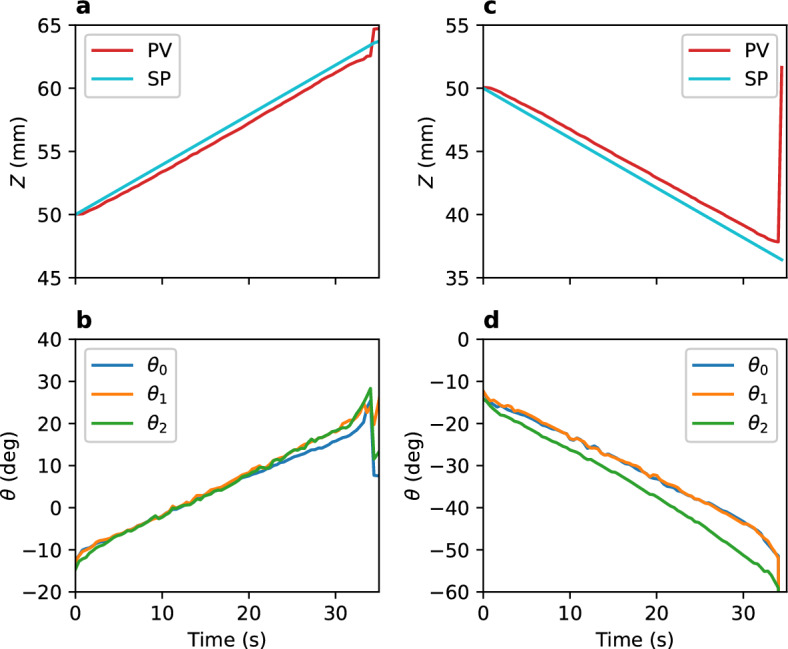
Feedback control for *Z* (**a**, **b**) from 50 to 65 mm and (**c**, **d**) from 50 to 35 mm with a constant velocity of 0.4 mm/s (*X* =  *Y* = 0). (**a**, **c**) indicate *Z* position. (**b**, **d**) show the magnet angles for (**a**, **c**), respectively.

**Figure 5:**
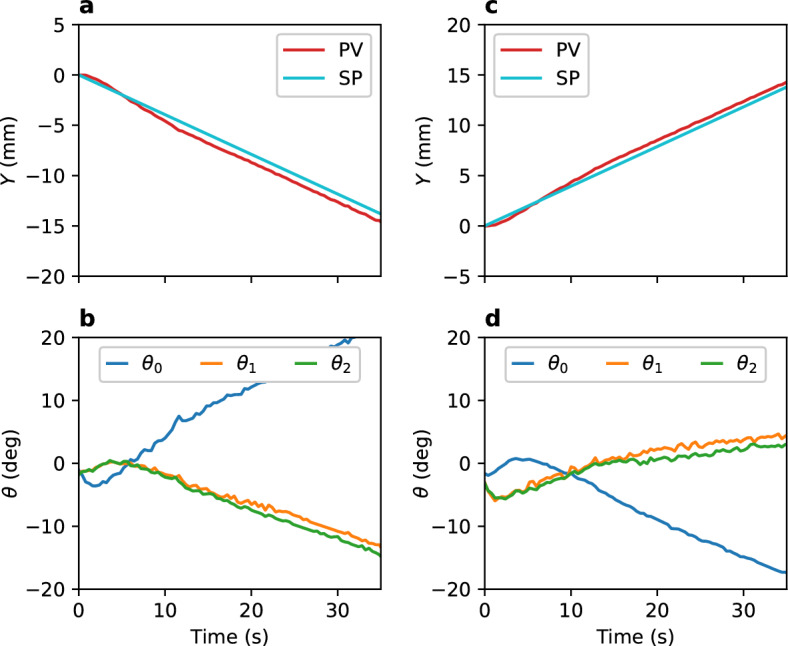
Feedback control for *Y* (**a**, **b**) from 0 to − 15 mm and (**c**, **d**) from 0 to 15 mm with a constant velocity of 0.4 mm/s (*Z* = 55 mm). (**a**, **c**) indicate *Y* position. (**b**, **d**) show the magnet angles for (**a**, **c**), respectively.

The control principles discussed in the previous section were examined using the magnetic motion control system. Figure 4 shows the feedback control results for *Z* position from 50 to 65 mm and 50 mm to 35 mm. The controllable range is approximately between *Z* = 38 and 63 mm. In this range, PV lags SP, and the magnet angles vary monotonically. Figure 5a and b show the feedback control results in the *XY* direction. The SP of *Y* was varied from 0 to − 15 mm. The PV of *Y* lags the SP until approximately 5 s, after which the situation reverses. Magnet 0 rotates in the negative direction at first to move the magnetic object in the – *Y* direction; however, it reverses at approximately 5 s because the error (difference between PV and SP) reverses in sign. This behavior satisfies the desired motion of the stable point in the – *Y* direction. Magnets 1 and 2 rotate in a direction opposite to that of magnet 0 to maintain the *Z* position. For movement in the +  *Y* direction, the situation is completely opposite, as shown in Fig. 5c and d. Thus, the control principles are found to be effective.

**Figure 6:**
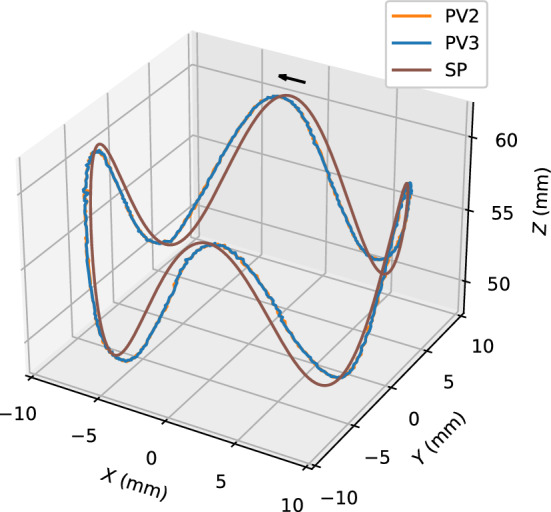
Trace for a circular path superimposed on a sine wave. (Supplementary Video S1, Supplementary Fig. S3). Maximum speed of the SP is 0.49 mm/s. PV2 and PV3 denote the PVs for the second and third laps.

**Figure 7:**
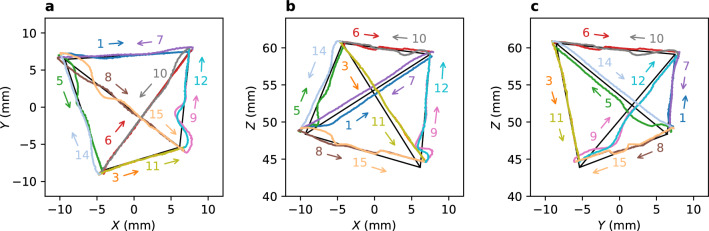
Trace for a tetrahedral path. (Supplementary Video S2, Supplementary Fig. S4). Projections in (**a**) *XY*, (**b**) *XZ*, and (**c**) *YZ* planes. Tracing order for the sides of the tetrahedron (denoted as numbers) is randomly selected. Some trajectories are omitted for clarity. Maximum speed of the SP is 0.33 mm/s.

Two 3D paths were examined. Figure 6 shows the results for a circular path superimposed on a sine wave. The magnetic object tracks the SP well, although PV lags SP throughout the path. The trajectories of the second and third laps coincide completely. Figure 7 shows the results for a tetrahedral path. The tetrahedral path was found to be more difficult than the circular path. In particular, after passing through the corners where the SP changed abruptly, the magnetic object did not follow the SP path. The deviation also increased at low *Z* positions.

## Discussion

**Figure 8:**
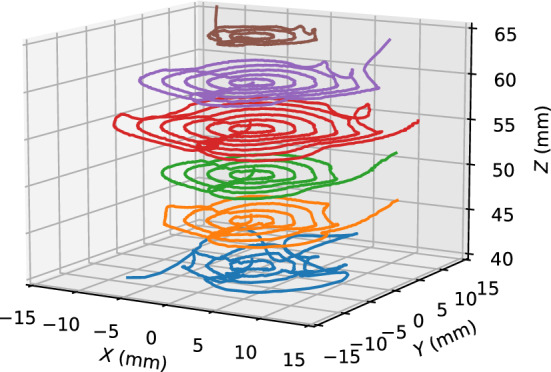
Feedback control for circular paths. Radius is increased until the magnetic object is out of control. Speed of the SP increases with the radius; it is 0.44 mm/s for a radius of 10 mm.

The experimental results presented in Figs. 4 and 7 suggest that the behavior of the magnetic object depends on the magnet positions; moreover, the controllable area is limited. This was examined using circular paths, as shown in Fig. 8. The radius was increased until the magnetic object was out of control. The maximum radius of the controllable area was 12 mm at *Z* = 55 mm. With increasing or decreasing *Z* position, the controllable area degraded.

When the magnetic object was out of control, its north pole inclined toward the center (*X* = *Y* = 0), as depicted in the torque maps in Fig. 3c and d. This enhanced outward movement, and finally, the north pole pointed downwards. At a point far away from the *Z*-axis or at a low *Z* position, the torque was strong; such an event frequently occurred because rotating magnets are in close proximity. However, at a high *Z* position, the response of the magnetic object to the angle of the rotating magnets was weak, and the control output tended to increase. This can also result in the magnetic object to turn over due to a large torque. To increase the controllable area, the inclination angle (*θ*) of the magnetic object must be limited.

Petruska et al.^19^ suggested that the minimum number of magnets required for 3-DOF control is four. Essentially, three magnets would be sufficient; however, an additional magnet is required to align the direction of the controlled magnet. Otherwise, a nonmagnetic restoring force, *e.g.*, gravity, can serve as the fourth magnet. As shown in Fig. 2, the magnetic force was exerted both downward and upward in the proposed system, which is contrary to the above claim. However, the feedback process indirectly aligns the north pole of the magnetic object, and it may serve as the fourth magnet. This topic requires further investigation.

Monitoring the position using cameras was crucial in the feedback control. However, this is impossible in cases where the magnetic object is invisible, such as in the human body. Xu et al.^5^ suggested alternatives such as fluoroscopy and X-ray. Such methods are worthy of further investigation. If the angles of the actuator magnets to propel the magnetic object in any direction or place it at any position are predicted, feedforward control can be performed without sensing the position of the magnetic object^3^. Such predictions may be realized with evolutionary computation including the covariance matrix adaptation evolution strategy^22^.

Several aspects of the presented system can be improved: for example, feedback gain optimization, scale-up for control in a larger area, and scale-down for control of fine particles. In principle, the viscosity of the solution can be decreased; moreover, levitation in air may be possible in an ultimate case.

## Methods

The magnetic force and torque acting on the magnetic object were calculated using Eqs. (1) and (2). The magnetic field was calculated using a 3D FEM simulation software tool, Femtet (Murata Software). The internal magnetic flux density of magnets 0–2 was set to 1.4 T^22^. The field gradient was calculated using the change in the magnetic field at points located at a distance of 1 mm from the point of interest.

The magnetic object was a cylindrical (φ 3 mm × 3 mm) ferrite magnet magnetized in the longitudinal direction. The magnetic moment, measured using a vibrating sample magnetometer, was 5.9 × 10^–3^ A m^2^. It was buried in a plastic sphere of 3-mm radius; a plastic rod of 6-mm length was attached to the sphere to detect the direction. The mass of the magnetic object was 220 mg (gravity was 2.2 mN), the volume was 130 mm^3^, and the buoyancy was estimated to be 1.3 mN based on Archimedes’ principle.

For the demonstration experiments, the magnetic object was immersed in polyvinyl alcohol and borax aqueous solution to impede movement. The viscosity of the solution, measured using a Brookfield viscometer (with spindle LV3 at 12 rpm), was 1.6 Pa s. A thread was attached to the magnetic object to facilitate its placement at the starting point. It was sagged during motion control to avoid exerting force on the magnetic object. It was not used in the experiments with arbitrary paths (Figs. 6 and 7).

Supplementary Fig. S5 shows the block diagram of feedback control. It was constructed using LabVIEW (National Instruments) (Supplementary Information S1) with Vision Development Module. It was run at intervals of 0.4 s. The position of the magnetic object was monitored using two USB cameras that were pointed in the directions of *ϕ* =  − 45° and − 135° (see Fig. 1). The center of the acquired images was (*X*, *Y*, *Z*) = (0, 0, 55). The position of the magnetic object was recognized using the vision component. The *XY* positions of the SP and PV were converted to magnet coordinates (*m*_0_–*m*_2_), and the errors were used for the feedback. Proportional and integral controllers were set independently for *m*_0_–*m*_2_ and *Z* positions. However, the identical gains were used for the controllers; the proportional and integral gains were 7 deg/mm and 1.4 deg/mm s, respectively (positive for *Z* control and negative for *XY* control). Control inputs for the *m*_0_–*m*_2_ and *Z* positions were added to obtain manipulated values of *θ*_0_–*θ*_2_. Magnets 0–2 were rotated using geared stepper motors^23^, which were coupled using machine-cut brass parts and adhesive. This simple feedback scheme enables movement in the desired direction and stops at a point discussed in the finite-element method simulations section.

### Supplementary Information

Supplementary Figure 1.

Supplementary Figure 2.

Supplementary Figure 3.

Supplementary Figure 4.

Supplementary Figure 5.

Supplementary Table 1.

Supplementary Video 1.

Supplementary Video 2.

Supplementary Information 1.

## Data Availability

The datasets generated during the current study are available from the corresponding author on reasonable request.
