# Supplementary figures and images for: Three-dimensional motion control of an untethered magnetic object using three rotating permanent magnets
Source: Sci Rep. 2023 Oct 23;13:18052. doi: 10.1038/s41598-023-45419-2 (PMC10593945; doi:10.1038/s41598-023-45419-2)

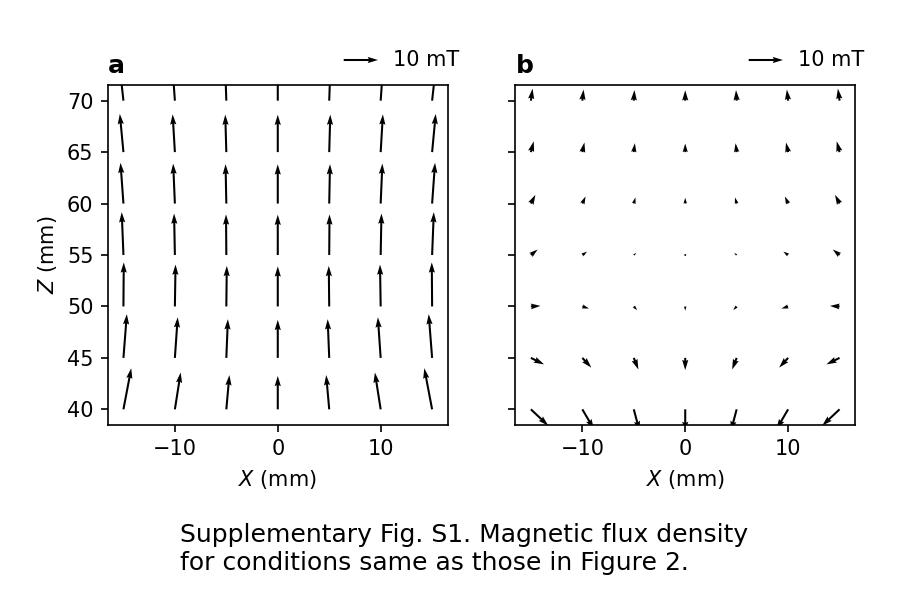

Supplement: Supplementary file 1 — Supplementary Figure 1. [file 41598_2023_45419_MOESM1_ESM.jpg]

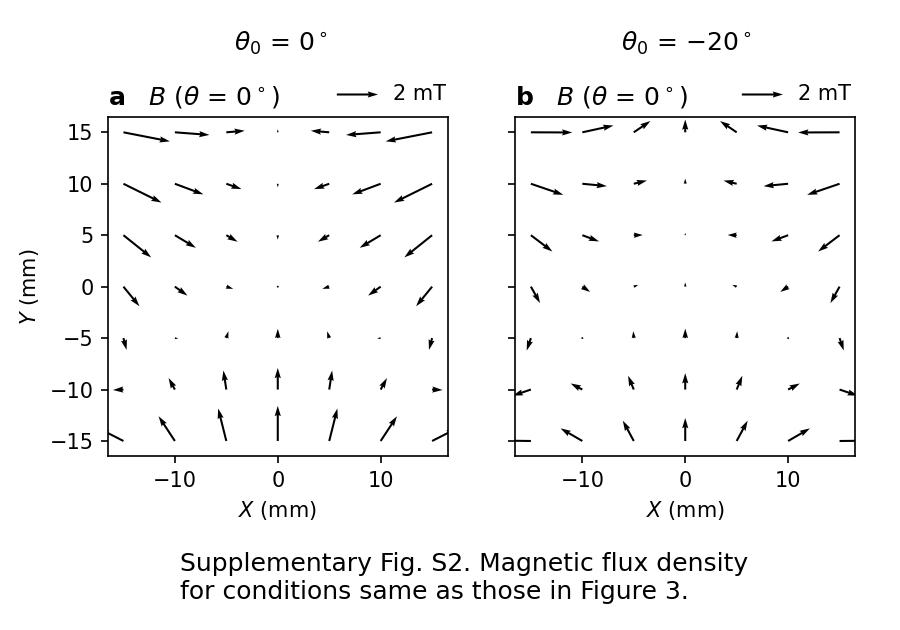

Supplement: Supplementary file 2 — Supplementary Figure 2. [file 41598_2023_45419_MOESM2_ESM.jpg]

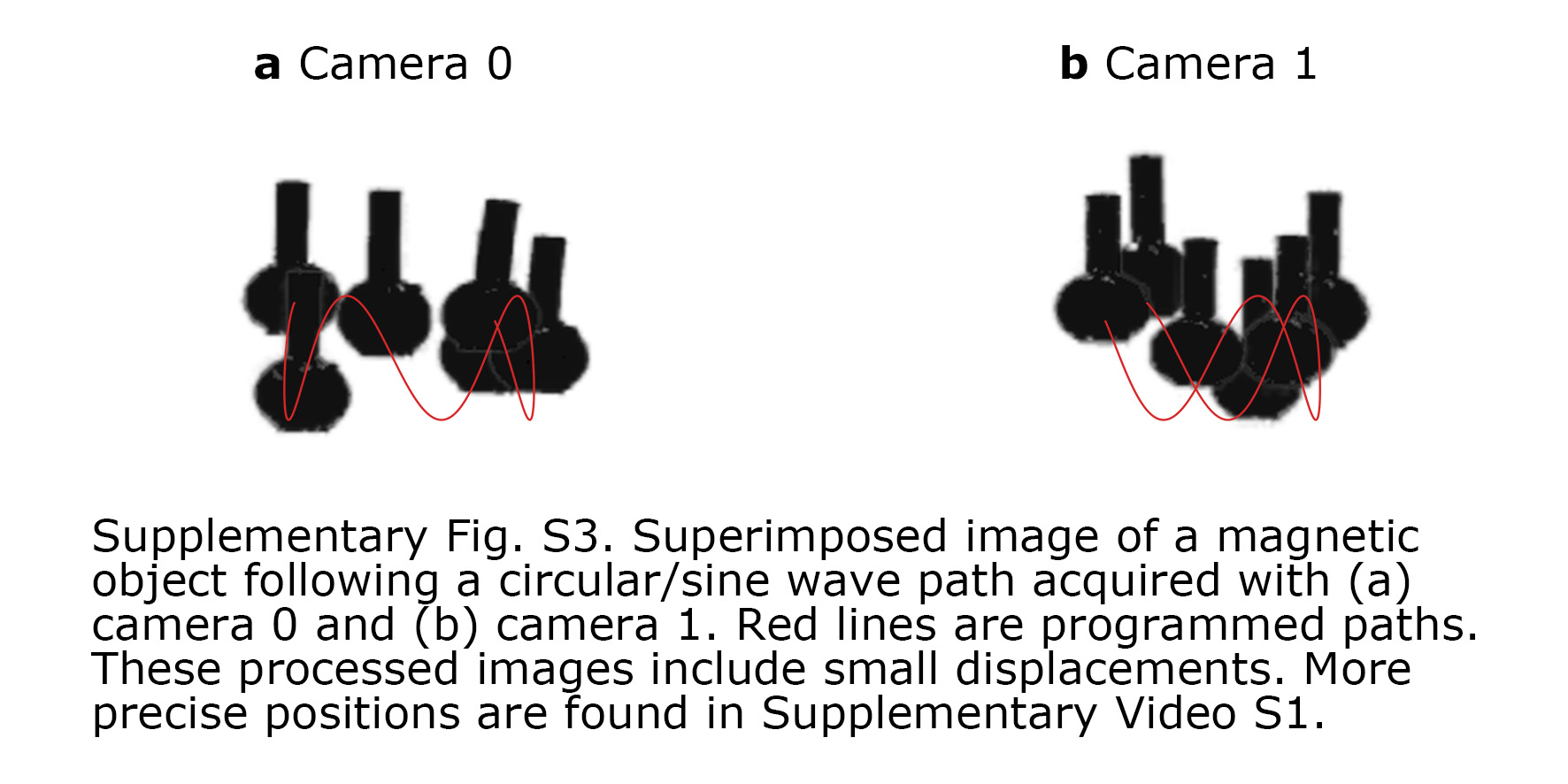

Supplement: Supplementary file 3 — Supplementary Figure 3. [file 41598_2023_45419_MOESM3_ESM.jpg]

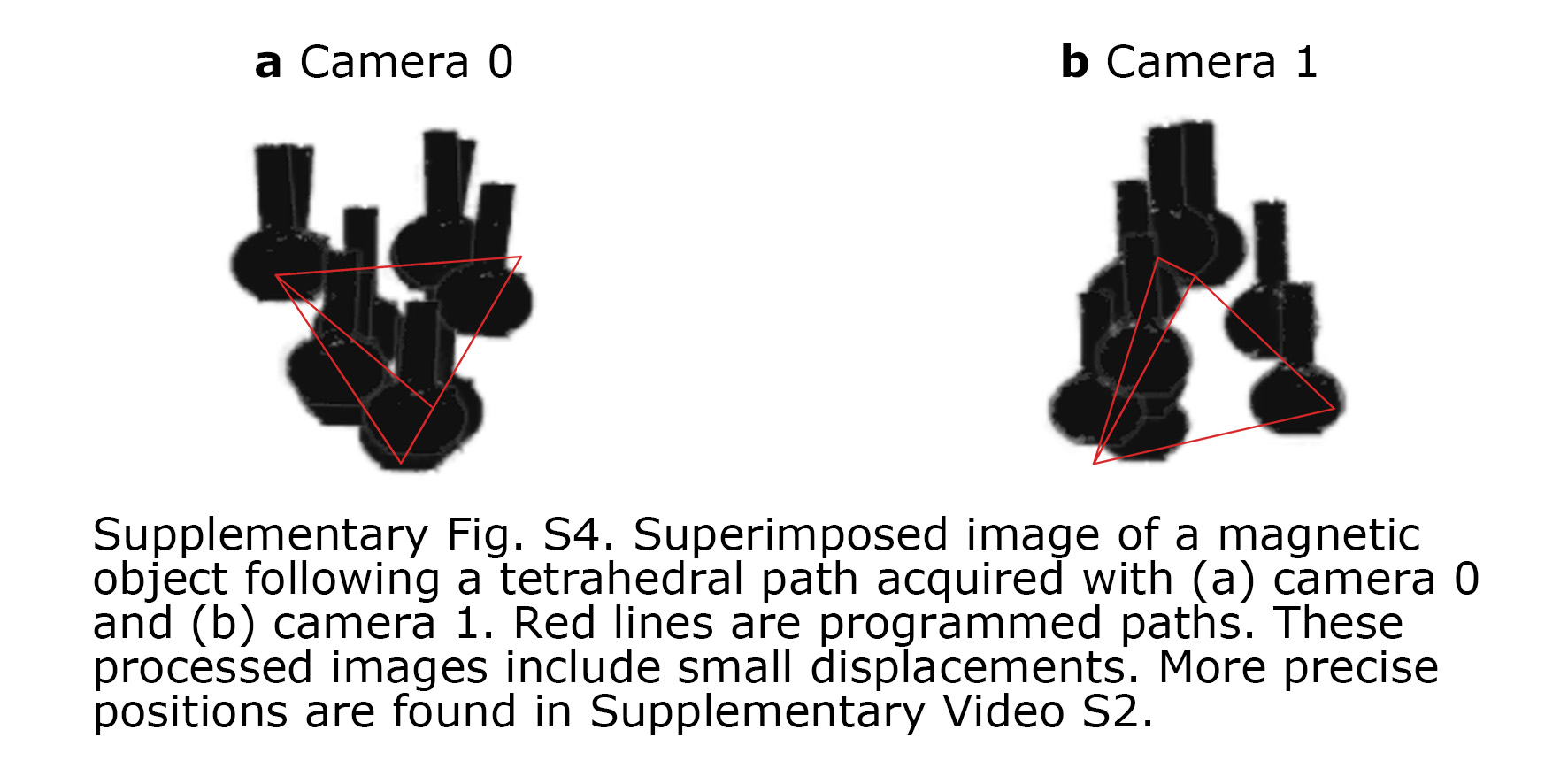

Supplement: Supplementary file 4 — Supplementary Figure 4. [file 41598_2023_45419_MOESM4_ESM.jpg]

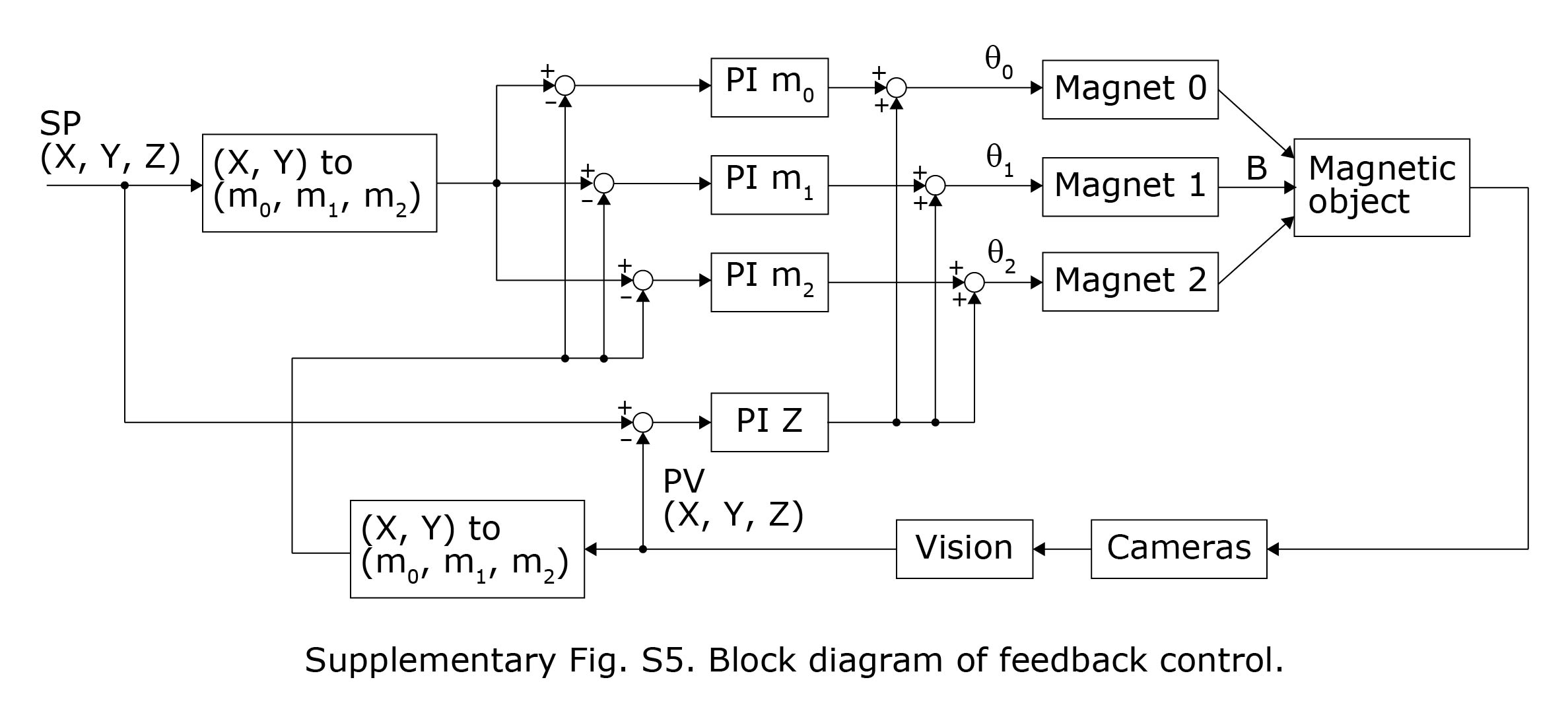

Supplement: Supplementary file 5 — Supplementary Figure 5. [file 41598_2023_45419_MOESM5_ESM.jpg]

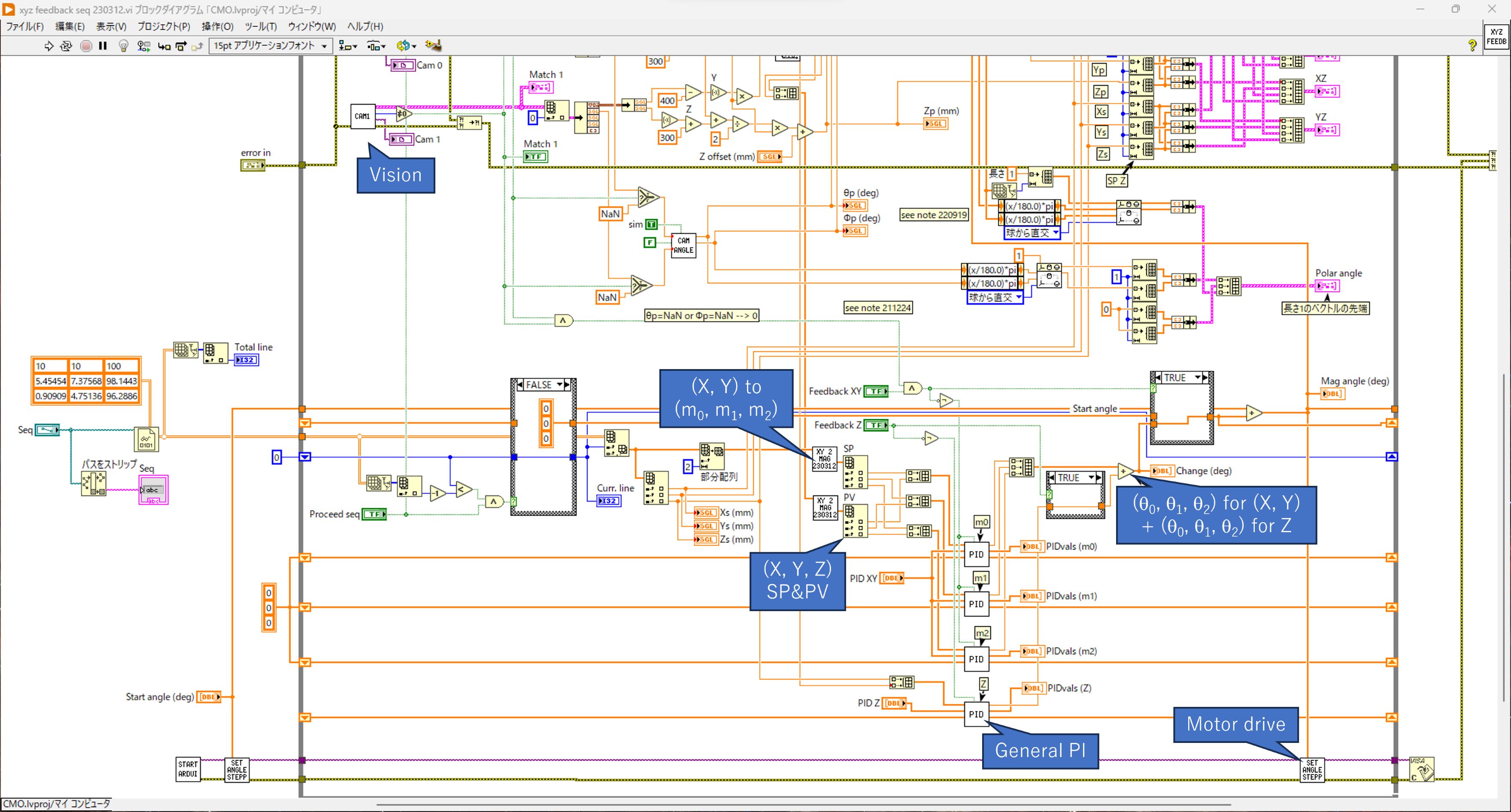

Supplement: Supplementary file 9 — Supplementary Information 1. [file 41598_2023_45419_MOESM9_ESM.zip › Code_S1/main block diagram.jpg]
