# Supplementary material for: Three-dimensional motion control of an untethered magnetic object using three rotating permanent magnets
Source: Sci Rep. 2023 Oct 23;13:18052. doi: 10.1038/s41598-023-45419-2 (PMC10593945; doi:10.1038/s41598-023-45419-2)
Supplement: Supplementary file 6 — Supplementary Table 1. [file 41598_2023_45419_MOESM6_ESM.pdf]

Supplementary Table S1. Demonstrations of magnetic motion control with permanent magnets.

Reference	DOF (position)	Workspace	Control principle	Number of actuator magnets	Motion of actuator magnets	Calculation
13	2	Open	Gradient	1	XY stage with motors	Not required
12	2	Closed	Gradient	2 Halbach cylinders	Manual rotation	Not required
3	2	Open	Gradient	4	Robotic arm	Not required
10, 17	3	Open	Gradient /Helical	1	Robotic arm	Required
11	3	Closed	Gradient /Helical	8	Rotation by motors	Required
Present study	3	Open	Gradient	3	Rotation by motors	Not required
